# Antiepileptic drug-induced psychosis associated with *MTHFR* C677T: a case report

**DOI:** 10.1186/s13256-019-2188-3

**Published:** 2019-08-12

**Authors:** Masaru Shimura, Hikari Yamada, Hidekuni Takahashi, Naoto Yamada, Soken Go, Gaku Yamanaka, Hisashi Kawashima

**Affiliations:** 1 Department of Pediatrics, Tokyo Medical University Ibaraki Medical Center, 3-20-1 Chuo, Ami-machi, Inashiki-gun, Ibaraki, 300-0395 Japan; 2 Department of Pediatrics and Adolescent Medicine, Tokyo Medical University, 6-7-1 Nishishinjuku, Shinjuku-ku, Tokyo, 160-0023 Japan

**Keywords:** 5,10-Methylenetetrahydrofolate reductase, MTHFR, C677T, Schizophrenia, Psychosis, Antiepileptic drugs, Hyperhomocysteinemia, Vitamin deficiency, Folate

## Abstract

**Background:**

Various antiepileptic drugs can potentially cause psychiatric side effects in patients with epilepsy, but the precise mechanism of these actions remains unknown. In recent years, the common polymorphism C677T in the 5,10-methylenetetrahydrofolate reductase (*MTHFR*) gene has attracted attention for its role in the onset of psychiatric diseases. MTHFR and several vitamins (as cofactors) are crucial for remethylation of homocysteine via folate and homocysteine metabolism. We report a case of a Japanese patient who presented with reversible schizophrenia-like symptoms during antiepileptic drug therapy.

**Case presentation:**

Our patient had frontal lobe epilepsy and had been treated with several antiepileptic drugs since the age of 13 years. He developed auditory hallucinations and multiple personalities at 17 years of age, several months after the initiation of phenytoin and phenobarbital, despite these antiepileptic drugs being used within the therapeutic ranges. Genetic analysis revealed that he was homozygous for the C677T polymorphism of *MTHFR*. Hyperhomocysteinemia, hypomethionemia, and multiple vitamin deficiencies, including folate, riboflavin, and pyridoxal, were identified at the age of 23 years. Vitamin supplementation and alteration of the antiepileptic drugs improved his psychotic symptoms. Multiple vitamin deficiencies with homozygous *MTHFR* C677T should be considered in patients presenting with schizophrenia-like symptoms during antiepileptic drug therapy.

**Conclusions:**

To the best of our knowledge, this is the first report of antiepileptic drug-induced psychosis associated with homozygous C677T and multiple vitamin deficiencies. Our findings will contribute to the elucidation of the pathogenesis of the psychiatric side effects of antiepileptic drugs and lead to improved medical management for patients with epilepsy.

## Background

**Fig. 1:**
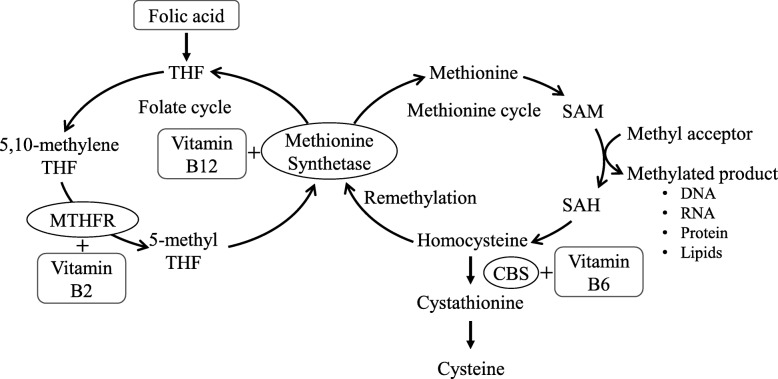
Folate pathway and homocysteine metabolism. *CBS* Cystathionine β-synthetase, *SAH S*-adenosylhomocysteine, *THF* Tetrahydrofolate

The riboflavin-dependent enzyme 5,10-methylenetetrahydrofolate reductase (MTHFR; MIM 607093; EC 1.5.1.20) converts 5,10-methylenetetrahydrofolate to 5-methyltetrahydrofolate during folate and homocysteine metabolism (Fig. 1) [1, 2]. Several vitamins, such as riboflavin (vitamin B_2_), pyridoxal (vitamin B_6_), and cobalamin (vitamin B_12_), play important roles as cofactors in the metabolism of homocysteine. The remethylation of homocysteine is a pivotal reaction, because adequate concentrations of methionine are required for *S*-adenosylmethionine (SAM) to act as a methyl donor for the methylation of deoxyribonucleic acid (DNA), ribonucleic acid (RNA) and other small molecules [3, 4].

Severe MTHFR deficiency, a rare autosomal recessive disorder, results in defects in the remethylation of homocysteine. Early-onset MTHFR deficiency manifests as feeding difficulties, hydrocephalus, and apnea during infancy, whereas adult-onset MTHFR deficiency manifests as seizures, cognitive impairment, and behavior or psychiatric disorders [2]. *MTHFR* C677T polymorphism (rs1801133) causes thermolability leading to a mild reduction in enzyme activity. Although this polymorphism is not associated with severe MTHFR deficiency, it is related to various vascular and neuropsychiatric diseases [2, 5, 6].

Various antiepileptic drugs (AEDs) can cause psychiatric side effects, but the pathogenesis of these effects has not been fully elucidated [7]. We describe a patient with homozygous *MTHFR* C677T who presented with reversible psychosis during AED therapy. To the best of our knowledge, this is the first report of AED-induced psychosis associated with *MTHFR* C677T and multiple vitamin deficiencies.

## Case presentation

**Fig. 2:**
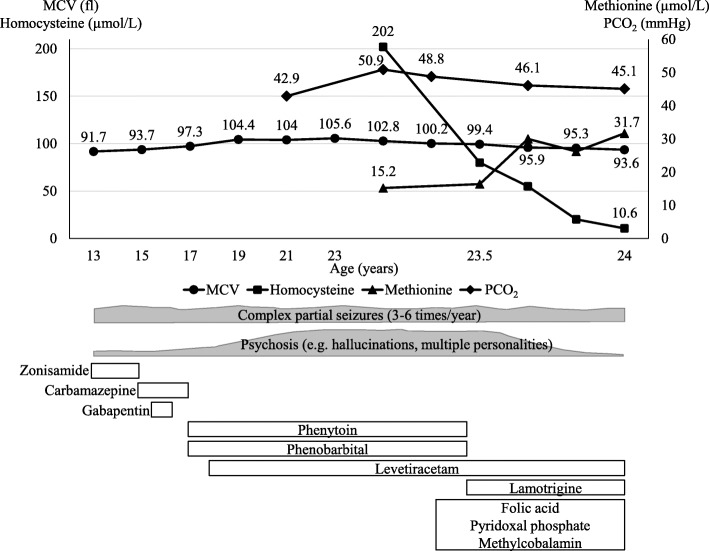
Clinical course of the patient. The changes in the mean corpuscular volume, serum homocysteine levels, plasma methionine levels, and venous partial pressure of carbon dioxide levels with neuropsychiatric symptoms and treatments are indicated

The patient was a Japanese male who was born at 38 weeks of gestation as the first child to nonconsanguineous parents. His newborn screening tests had yielded normal findings. His development and growth were uneventful, and his diet was balanced. He had no family history of coronary artery disease, stroke, or neuropsychiatric diseases. He presented with abnormal behavior such as running into the street, sudden sleep attacks, and personality changes from the age of 10. His first tonic seizure occurred at the age of 13. Electroencephalogram (EEG) findings revealed sharp wave runs in the frontal region, and he was diagnosed with frontal lobe epilepsy. His clinical course is depicted in Fig. 2. Although treatment with zonisamide, carbamazepine, and gabapentin was initiated, his seizures were not controlled. Treatment with phenytoin and phenobarbital was initiated at the age of 17. Subsequently, regular follow-up blood tests revealed that his mean corpuscular volume (MCV) was elevated to > 100 fl without a decline in hemoglobin levels. Six months after the introduction of treatment with phenytoin and phenobarbital, the patient reported auditory hallucinations, and multiple personalities appeared. Although he was treated with phenytoin, phenobarbital, and levetiracetam within the relevant therapeutic ranges for 6 years, epileptic seizures occurred repetitively with EEG abnormalities, and his schizophrenia-like symptoms gradually progressed. He and his parents were referred to a psychiatrist, but the patient refused treatment. He went on to study at a university without experiencing any problems; however, he abruptly left the university during his fourth year. He subsequently exhibited slow movement, intention tremor, impairment of short-term memory, and dysgraphia. He remained at home without a job after graduating from the university.

**Fig. 3:**
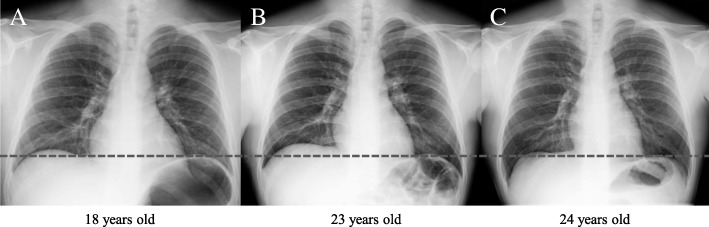
Changes in the diaphragm position on chest x-rays. The *horizontal dotted line* indicates the left diaphragm position. **b** A slight elevation of the right diaphragm can be observed relative to its position in (**a**). **c** Chest x-ray obtained after normalization of the serum homocysteine level illustrating the right diaphragm in a lower position than in (**b**)

Upon admission to our hospital due to a tonic seizure at the age of 23, the patient underwent a detailed examination. He had no gingival overgrowth but did have macrocytosis without anemia (hemoglobin, 16.8 g/dl; MCV, 102.8 fl). We observed mild elevation of the partial pressure of carbon dioxide (PCO_2_) and bicarbonate without acidemia in the venous blood, suggesting chronic CO_2_ retention (pH, 7.386; PCO_2_, 50.9 mmHg; bicarbonate, 29.2 mmol/L). The blood concentrations of all AEDs were within the relevant therapeutic ranges (phenytoin, 13.35 μg/ml [therapeutic range, 10–20 μg/ml]; phenobarbital, 20.81 μg/ml [10–35 μg/ml]; levetiracetam, 15.8 μg/ml [12–46 μg/ml]). The patient’s serum levels of folate, riboflavin, and pyridoxal decreased to below-normal ranges, and his cobalamin level decreased to the lower limit of the normal range (folate, 1.6 ng/ml [normal range, 3.6–12.9 ng/ml]; riboflavin, 3.6 μg/dl [12.8–27.6 μg/dl]; pyridoxal, 3.9 ng/ml [6.0–40.0 ng/ml]; cobalamin, 275 pg/ml [233–914 pg/ml]). Plasma amino acid analysis revealed a decrease in the methionine level and a marked elevation in the plasma homocysteine level, whereas other essential amino acids were within normal ranges (methionine, 15.2 μmol/L [18.9–40.5 μmol/L]; homocysteine, 202 μmol/L [5.0–15.0 μmol/L]). Urine organic acid analysis revealed no abnormal findings, including methylmalonic acid. A chest x-ray demonstrated a slight elevation of the right diaphragm relative to the findings of a previous x-ray (Fig. 3a, b). Brain magnetic resonance imaging revealed no abnormalities. Genetic analysis identified three homozygous polymorphisms in the *MTHFR* gene (NM_005957.4): c.665C>T (p.Ala222Val; also known as C677T), c.1166 + 31C>T, and c.1305C>T (p.Phe435=). Supplementation with folic acid (15 mg/day), pyridoxal phosphate (30 mg/day), and methylcobalamin (1.5 mg/day) was started, and phenytoin and phenobarbital were switched to lamotrigine. Thereafter, the patient’s MCV decreased to < 100 fl, and his methionine level was normalized to the vitamin levels. PCO_2_ and bicarbonate levels in the venous blood continued to decrease, which was accompanied by a normalization of the diaphragm position (Figs. 2 and 3c). The patient’s auditory hallucinations, multiple personalities, lethargy, and other neuropsychological symptoms improved as serum homocysteine levels were reduced to within normal ranges with supplementation. However, his seizure attacks still occurred once per month.

## Discussion and conclusions

The presented case highlighted two important issues: (1) Long-term administration of AEDs to patients with homozygous *MTHFR* C677T can cause hyperhomocysteinemia and schizophrenia-like psychosis, similar to that which is observed in adult-onset MTHFR deficiency; and (2) AED-induced psychosis is associated with homozygous C677T and multiple vitamin deficiencies.

Our patient had three homozygous polymorphisms in *MTHFR*. The allele frequencies of c.665C>T (also known as C677T), c.1166 + 31C>T, and c.1305C>T in East Asian populations are reported to be 0.2903, 0.7592, and 0.9991, respectively, according to the Genome Aggregation Database. The associations of *MTHFR* C677T with various diseases have been well documented. In particular, the correlation between this polymorphism and the risk of coronary artery disease, stroke, and neuropsychiatric diseases has received considerable attention in recent years [6, 8]. Polymorphism of this gene is also a risk factor for folate deficiency, which results in neural tube defects [1]. The C677T allele frequency differs greatly among ethnic groups, with a T allele frequency ranging from 1% in African Americans to 30% in Japanese and Europeans [9, 10]. In a Japanese population, the C677T allele frequency was very high, and the frequency of homozygous genotypes was 11% [11]. The residual enzyme activity from cultured fibroblasts of patients with severe MTHFR deficiency was < 20%, and the age of onset and severity of the disease correlated with the enzyme activity [12, 13]. *MTHFR* C677T also reduced the enzyme activity, resulting in a 30% decrease in heterozygotes and a 65% decrease in homozygotes [14]. In this context, the common polymorphism C677T has not been considered as a cause of severe MTHFR deficiency [2, 15].

Folate, riboflavin, pyridoxine, and cobalamin deficiencies, as observed in our patient, can cause hyperhomocysteinemia because these vitamins are involved in folate and homocysteine metabolism [16]. Elevation of MCV was observed soon after the initiation of phenytoin and phenobarbital supplementation, suggesting a folate or cobalamin deficiency. Folate deficiency has been reported to contribute to the onset of psychiatric diseases such as schizophrenia and bipolar disorder [8]. Furthermore, a causal relationship between homocysteine levels affected by C677T and schizophrenia was revealed in a meta-analysis [17]. Both folate deficiency and *MTHFR* C677T contribute to impaired remethylation of homocysteine to methionine, resulting in a decrease in SAM. It is plausible that SAM deficiency causes abnormal methylation of DNA, proteins, and neurotransmitter and DNA strand breaks, leading to psychiatric diseases [18]. Homocysteine-reducing strategies using folic acid, pyridoxine, and vitamin B_12_ have been demonstrated to improve clinical symptoms in schizophrenia [19].

The psychiatric side effects of various AEDs have been described in many studies over several decades [7]. Although the forced normalization of EEG is associated with AED-induced psychosis, our patient did not exhibit EEG normalization during his clinical course. Phenytoin-induced schizophrenia-like psychoses, the mechanism of which remains poorly understood, may be dose-dependent, but serum levels in our patient were maintained within the therapeutic ranges [20]. However, a decrease in MTHFR activity by phenytoin treatment at therapeutic plasma levels was observed in mice [21]. Enzyme-promoting AEDs such as phenytoin and phenobarbital are known to cause riboflavin and folate deficiencies via induction of cytochrome P450 [16, 22]. It has been reported that phenytoin decreases serum pyridoxal levels, leading to hyperhomocysteinemia [23]. Furthermore, phenytoin administration in patients with epilepsy who have the *MTHFR* TT genotype markedly increased plasma homocysteine levels compared with levels induced by administration of other AEDs [24]. Thus, the marked elevation of plasma homocysteine in our patient may have been caused by multiple factors, namely the common homozygous C677T; the folate, riboflavin, and pyridoxal deficiencies; and the enzyme-promoting AED.

The patient had mildly increased CO_2_ levels and slight elevation of the right diaphragm, which improved with normalization of serum homocysteine levels. Neonates and young infants with severe MTHFR deficiency often present with respiratory complications such as apnea and phrenic nerve palsy [2, 25]. Although the precise mechanism of these respiratory complications remains poorly understood, it has been presumed to result from central and peripheral neurological impairment; this can be attributed to SAM deficiency, which is involved in dysmyelination [4, 26]. To our knowledge, no clinical studies have documented *MTHFR* C677T-related respiratory symptoms. C677T-associated hyperhomocysteinemia should be considered as a differential diagnosis in patients presenting with respiratory symptoms during AED therapy.

To the best of our knowledge, this is the first report of AED-induced psychosis associated with homozygous C677T and multiple vitamin deficiencies, which was similar to that observed in adult-onset MTHFR deficiency. The long-term administration of AEDs in patients with the homozygous *MTHFR* c.677C>T genotype can cause folate, riboflavin, pyridoxal, and cobalamin deficiencies, leading to hypomethionemia and hyperhomocysteinemia, even when the administered AEDs are within the therapeutic ranges. In cases of schizophrenia-like symptoms during AED therapy, vitamin deficiencies should be suspected, which can be treated with vitamin supplementation and different AEDs. Further reports are needed to confirm these findings and elucidate the pathogenesis of AED-induced psychosis.

## Data Availability

The datasets supporting the conclusions of this article are included within the article.

## Abbreviations

AED: Antiepileptic drug
EEG: Electroencephalogram
MCV: Mean corpuscular volume
MTHFR: 5,10-Methylenetetrahydrofolate reductase
PCO_2_: Partial pressure of carbon dioxide
SAM: *S*-adenosylmethionine
